# Is there a difference in body size dissatisfaction between the patients with obesity seeking and not seeking treatment for obesity?

**DOI:** 10.1186/s12889-021-11771-z

**Published:** 2021-09-26

**Authors:** Wojciech Gruszka, Aleksander J. Owczarek, Mateusz Glinianowicz, Monika Bąk-Sosnowska, Jerzy Chudek, Magdalena Olszanecka-Glinianowicz

**Affiliations:** 1 Health Promotion and Obesity Management Unit, Department of Pathophysiology, Medical Faculty in Katowice, Medical University of Silesia, Katowice, Poland; 2 Pathophysiology Unit, Department of Pathophysiology, Medical Faculty in Katowice, Medical University of Silesia, Medyków Street, 18 20 40-752 Katowice, Poland; 3 Department of Psychology, Chair of Social Sciences and Humanities, School of Health Sciences in Katowice, Medical University of Silesia, Katowice, Poland; 4 Department of Internal Medicine and Oncological Chemotherapy, Medical Faculty in Katowice, Medical University of Silesia, Katowice, Poland

**Keywords:** Perception of nutritional status, Body size satisfaction, Seeking treatment for obesity, Obesity, Motivation for weight loss

## Abstract

**Background:**

Various factors motivate people to undertake treatment for obesity. Among others they include health benefits, willingness to please others, and dissatisfaction with one’s appearance. The present study aimed to assess body size dissatisfaction in patients with obesity seeking and not seeking treatment for obesity.

**Methods:**

Two-hundred-sixteen adult subjects (154 women, 62 men) including 80 people with BMI ≥ 30 kg/m^2^ starting treatment for obesity (BMI 35.7 ± 5.3 kg/m^2^) and 136 volunteers with obesity (BMI 34.7 ± 4.3 kg/m^2^) not seeking treatment for obesity, were enrolled. Body size satisfaction was assessed using the Figure Rating Scale adapted by Stunkard.

**Results:**

Patients with obesity starting obesity treatment had more often a high level of body size dissatisfaction than volunteers with obesity not seeking the treatment (*p* <  0.001). There was a significant difference in the distribution of body size dissatisfaction in women (*p* <  0.05), but not in men (*p* = 0.47).

**Conclusion:**

Patients with obesity, especially women, seeking obesity treatment more often represent a high level of body size dissatisfaction than volunteers with obesity not seeking treatment for obesity. This implies the need for public health campaigns to address negative attitudes or misconceptions about obesity and its treatment. Placing more attention and emphasis on body size dissatisfaction in more vulnerable women with obesity may help to define personal motivations and goals, strengthen the doctor-patient relationship and better adapt therapeutic strategies.

## Background

An increased incidence of overweight and obesity worldwide has been reported by numerous studies [1, 2]. Treatment rates of obesity and its complications seem to improve generally, however they are not clearly determined [3, 4]. External and internal factors influencing the decision about taking treatment of obesity are hardly known. Among others, family support, quality of life and willingness to improve appearance [5–7] were proved to be motivators to seek obesity treatment. With a contemporary growing prevalence of overweight and obesity, a better understanding of motivations for weight loss is needed. Identification of factors predisposing to undertake treatment of obesity will help to define better treatment strategies for health professionals. Moreover, the recognition of factors affecting motivation for weight loss treatment may help patients to delineate their personal goals, understanding realistic/unrealistic expectations, thus ameliorating results in terms of subjective well-being and quality of life.

It has been suggested that people with BMI ≥ 30 kg/m^2^ seeking obesity treatment have a different psychological profile than those who do not seek it [8]. A previously published study revealed that there were more women with moderate and severe depressive symptoms in the group starting treatment than in the group not seeking treatment for obesity (44.7 and 24.4%, respectively). Such a difference was not observed in males [9]. However, a more detailed characterisation of people willing to take treatment of obesity supervised by physicians and other medical professionals and subsequent interventions toward the improvement of treatment-seeking rate seems to be an important concern of public health. It seems that the psychological factors that motivate to start obesity treatment include body image dissatisfaction.

Body image is defined as an internal image of our appearance and self-esteem in how others see us. Gender, age, race, body weight and its changes, as well as socio-cultural factors, affected the creation of body image disturbances [10–19]. It was shown that body image disturbances are common, and more severe among women [10] and younger subjects, especially adolescents [11]. They were also observed among subjects above 70 years old, who mostly described themselves as “a little too big” (women) or “just the right size” (men) [12]. Self-perception of being overweight appears to be more common in Caucasians than in Afro or Latin Americans [20]. Among sociodemographic variables education level is associated with body image dissatisfaction. Cheung et al. [21] observed that overweight men and underweight women with lower education level were less likely to desire a slimmer body shape than their counterparts with high education level.

Some studies revealed that the frequency of body size dissatisfaction increased with BMI values [15–17]. Of note, the recent review indicated general improvements in body image following bariatric surgery [22]. Furthermore, individuals who underwent body contouring surgery after bariatric interventions reported improvements in various body image indices compared to post-bariatric patients who did not undergo contouring surgery [22]. In contrast, Bianciardi et al. [23] found no correlation between body image dissatisfaction and BMI among patients with extreme obesity. It has also been shown that body size dissatisfaction may be a cause for the development of depression in people with overweight or obesity, especially women [24, 25]. A high level of body image dissatisfaction can be also the main reason to seek and start obesity treatment.

Therefore, the present study aimed to assess body size dissatisfaction in patients with obesity seeking and not seeking treatment for obesity.

## Methods

Two hundred and sixteen adults with BMI ≥ 30 kg/m^2^ (154 women, 62 men), including 80 with obesity starting obesity treatment program (41.8 ± 11.9 years, BMI 35.7 ± 5.3 kg/m^2^) and 136 volunteers with obesity not seeking treatment for obesity (43.8 ± 12.3 years, BMI 34.7 ± 4.3 kg/m^2^) were enrolled. The patient seeking obesity treatment were recruited among patients of the Obesity Treatment Clinic, collaborating with the authors. The program offered by the Clinic, described below, was covered by the Health National Found as a standard medical care in this center at the time of this study. This 6-month group treatment consisted of regular meeting with the physician (specialist of internal medicine and obesitology), dietitian, psychologist and physiotherapist (every 2 weeks, 4 structured sessions of about 30 min). Cognitive-behavioural principles introduced by psychologist were used to assist the patient to overcome barriers of changing lifestyle. The physiotherapist’s counselling was aimed to promote regular aerobic training. Dietitian counselling was based on dietary self-monitoring records. The task of the physician was coordination and counselling on the obesity-related health condition. The first 5 months of the treatment was oriented towards behavioural weight loss, whereas the last two meetings shifted to focusing on maintaining healthy behaviours after the end of the program. No pharmacological interventions were applied. This treatment aimed to obtain a 5–15% reduction of body weight in this period, as recommended [26]. The participants did not have to pay for it. They signed up for the program themselves - information about the program was available on the Internet and in local newspapers.

The volunteers with obesity were recruited by co-authors of the paper, which are physicians in their out-patients clinics. The following two questions were posed: Have you ever tried professional treatment against obesity? Are you interested in this kind of treatment? Two negative answers resulted in the subsequent invitation of patients to the presented study. The recruitment rate was 81%. The reasons for visits to the out-patients clinics were various, excluding obesity. The inclusion criteria were: 1. age over 18 years, 2. stable body weight during the last 3-month period, 3. obesity diagnosis at last 10 years earlier. The exclusion criteria were: 1. secondary obesity (endocrine disorders like Cushing’s syndrome and genetic disorders like Turner syndrome or Prader–Willi syndrome), 2. mental illness in medical history (lifetime bipolar disorders, schizophrenia and current substance use disorders). The study was approved by the Bioethical Committee of the Medical University of Silesia in Katowice.

All patients were tested in the Health Promotion and Obesity Management Unit, Department of Pathophysiology, Medical Faculty in Katowice, Medical University of Silesia in Katowice. Anthropometrical measurements were done by authors who are physicians. Assessment of body image dissatisfaction was performed by authors who are psychologists. For people starting the obesity treatment program, measurements took place before the first group meeting of the program. Not seeking treatment for obesity volunteers after performing all procedures of the study were invited to participate in the obesity treatment program.

Body weight (without shoes, in light clothing, using the calibrated and certified electronic RADWAG balance, with an accuracy of 0.1 kg) and height (in an upright standing position, without shoes, with an accuracy of 0.5 cm, using an integral part of RADWAG balance) were measured. BMI was calculated using the standard formula. Assessment of nutritional status was based on BMI according to WHO criteria defining obesity as BMI ≥30 kg/m^2^ [27].

Body size satisfaction was assessed based on the Figure Rating Scale (FRS) adapted by Stunkard, as a difference between silhouette indicated as currently owned and silhouette indicated as desirable. FRS was administered as a hard copy. The following instruction was given: “mark the silhouette which is the most similar to yours” and “mark the silhouette which you desire to possess”. This scale has been standardized for use in patients with obesity and, as concluded in numerous studies, can be an appropriate tool to assess body image dissatisfaction [28–30]. Due to the use of the silhouettes, it does not need adaptation to the native language of studied subjects. The subjects, according to their gender, were asked to indicate a male or female figure [30].

### Statistical analysis

Statistical analysis was performed using the STATISTICA 12.0 PL software package (StatSoft, Cracow, Poland).

The results were presented as mean ± standard deviation and percentages for the data in nominal and ordinal scale. The assessment of distribution was based on the Shapiro-Wilk test. To compare groups of subjects the χ^2^ test and the two-way analysis of variance with contrast analysis were used. The multiple logistic stepwise backward regression analysis, adjusted to gender, was done to explain the role of cofactors on described relation between treatment, age, BMI and the level of body image dissatisfaction. The Hosmer-Lemeshow test was used to assess goodness-of-fit. Results were considered statistically significant with a *p*-value of less than 0.05.

### Results

A basic characteristic of the study group is presented in Table 1. There were no statistically significant differences between seeking and not seeking treatment for obesity groups in age, body weight and BMI.

**Table 1:** Study groups characteristics

	Subjects seeking treatment for obesity (*n* = 80)	Subjects not seeking treatment for obesity (*n* = 136)	p
Women [%]	91.6	59.6	< 0.001
Age [years]	41.8 ± 11.9	43.8 ± 12.3	p_gender_ = 0.55
Women	41.5 ± 11.6	46.3 ± 10.3	p_treatment_ = 0.91
Men	44.4 ± 15.5	40.3 ± 14.2	
Body weight [kg]	93.6 ± 14.7	98.4 ± 15.5	p_gender_ = 0.89
Women	92.0 ± 13.8	93.3 ± 14.0	p_treatment_ = 0.21
Men	110.3 ± 15.4	105.8 ± 14.7	
BMI [kg/m^2^]	35.7 ± 5.3	34.7 ± 4.3	p_gender_ < 0.001
Women	35.6 ± 5.5	35.1 ± 4.6	p_treatment_ = 0.61
Men	36.2 ± 3.1	34.2 ± 3.9	p_interaction_ = 0.35

In the all study group 29 (13.4%) subjects had high level of body size dissatisfaction (difference between silhouette currently possessed and desired ≥ 5), 95 (44.0%) - moderate (difference 3–4) and 92 (42.6%) - low (difference ≤ 2).

**Fig. 1:**
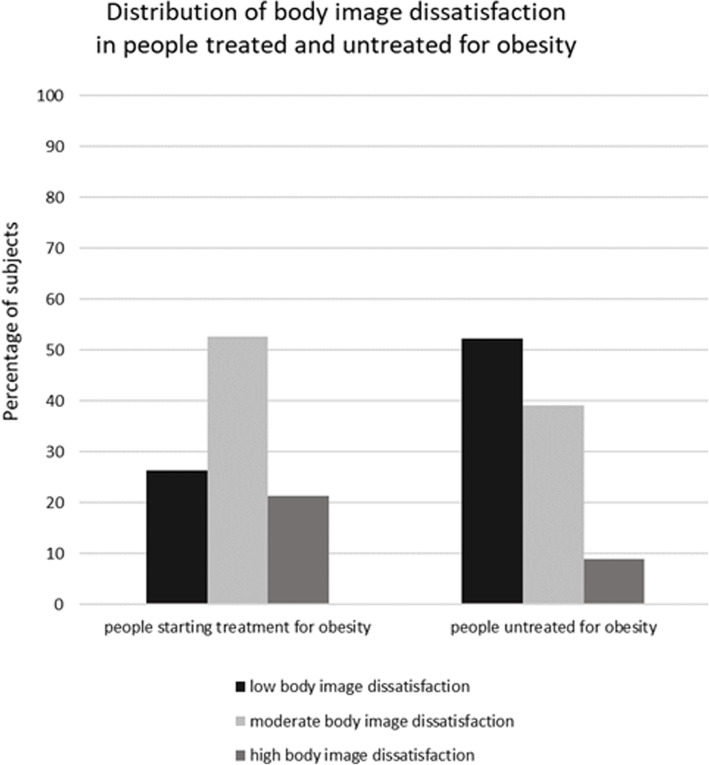
Distribution of body image dissatisfaction in subjects seeking and not seeking treatment for obesity

In the group of patients with obesity starting weight loss program 17 (21.3%) subjects had a high level of body size dissatisfaction, 42 (52.5%) moderate and 21 (26.2%) low. In the group of volunteers with obesity not seeking treatment for obesity, there were 12 (8.8%) subjects that had a high level of body size dissatisfaction, 53 (39%) - moderate and 71 (52.2%) - low (Fig. 1).

The high level of body size dissatisfaction significantly more often was observed among subjects starting obesity treatment than among volunteers with obesity not seeking treatment for obesity (χ^2^ = 15.9, *p* < 0.001).

**Fig. 2:**
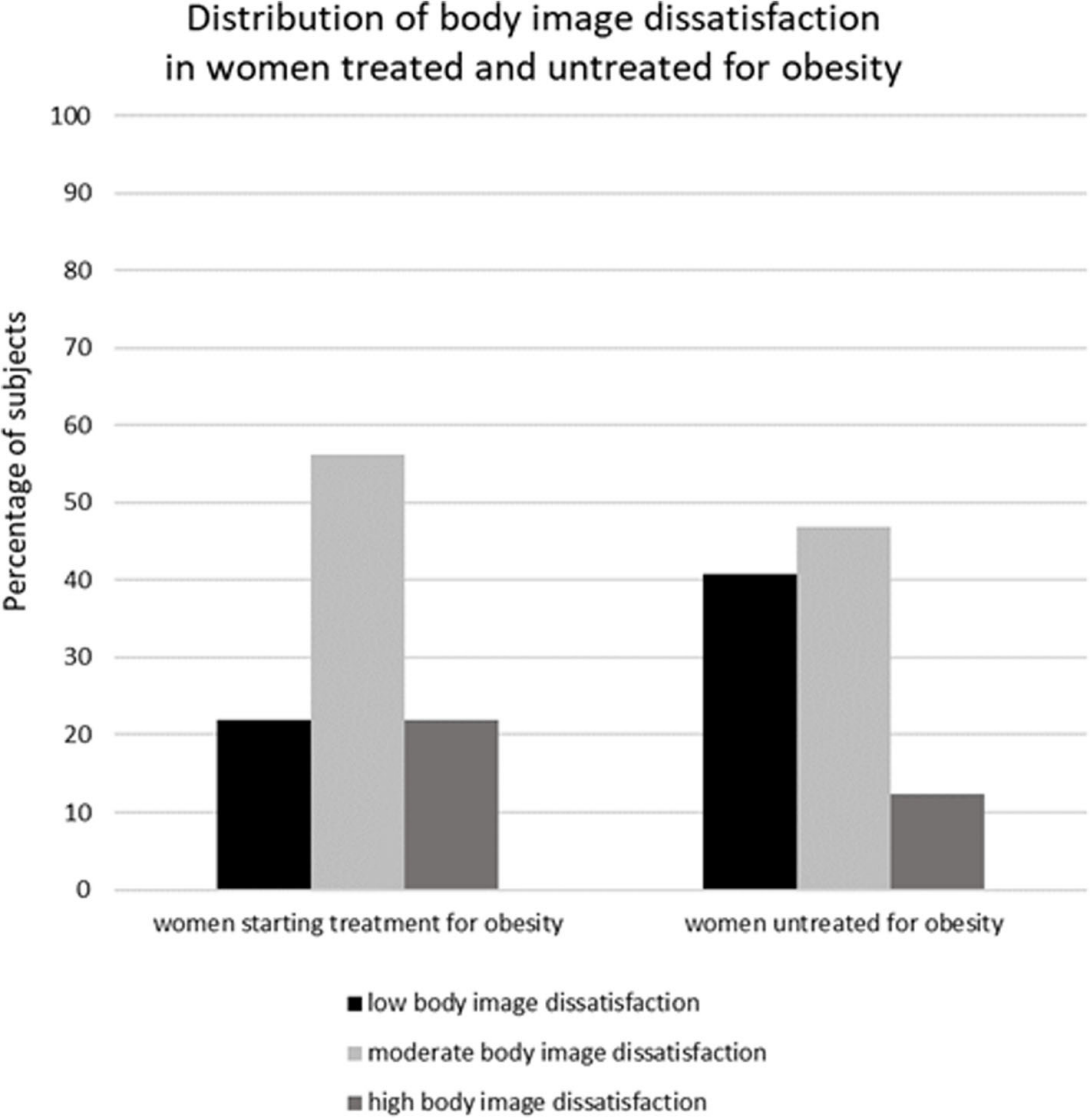
Distribution of body image dissatisfaction in women seeking and not seeking treatment for obesity

Among 73 women starting obesity treatment there were 16 (21.9%) subjects with the high level of body size dissatisfaction, 41 (56.2%) moderate and 16 (21.9%) low; and among not seeking treatment for obesity 10 (12.3%), 38 (46.9%) and 33 (40.8%), respectively (Fig. 2).

**Fig. 3:**
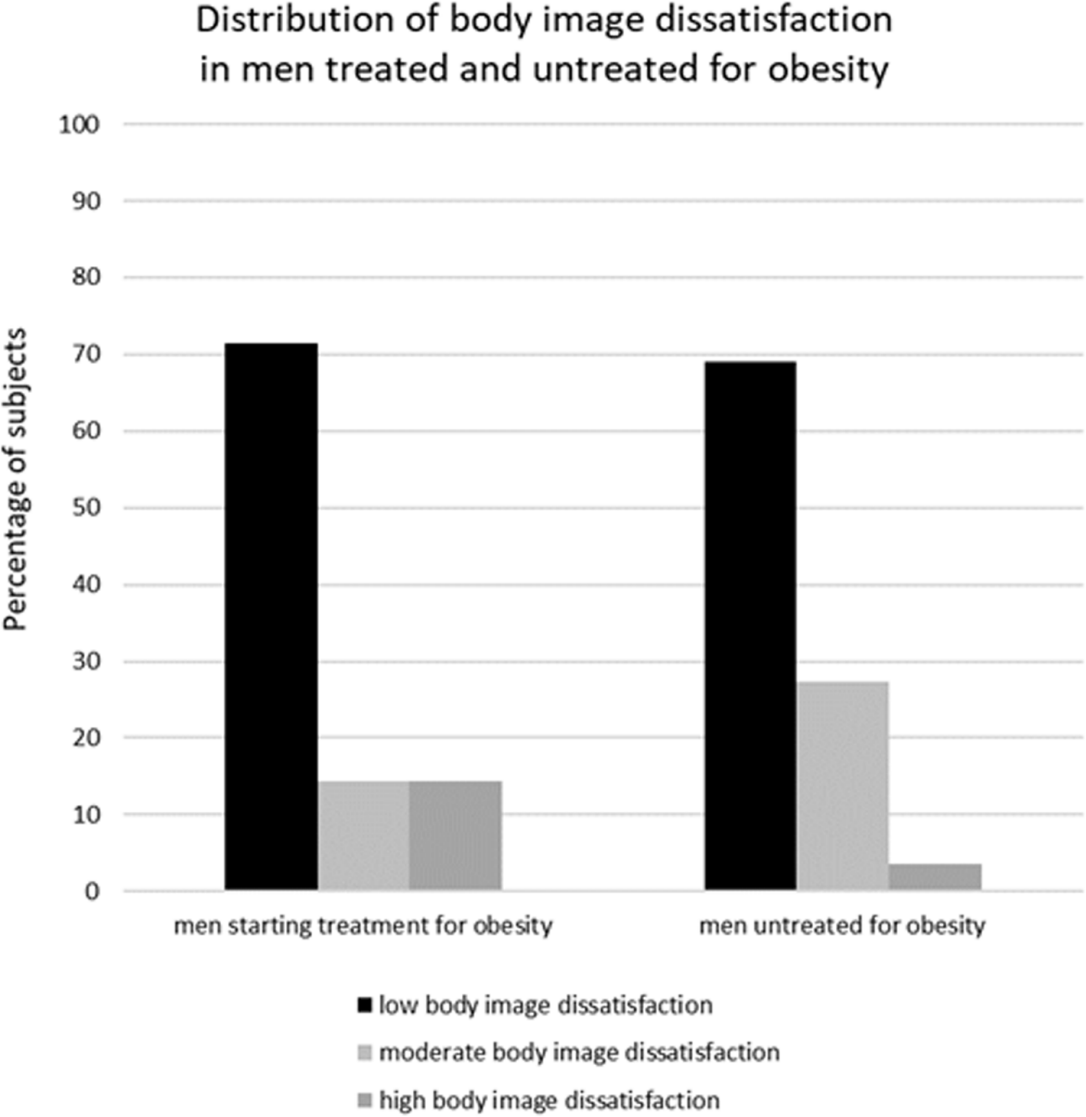
Distribution of body image dissatisfaction in men seeking and not seeking tratment for obesity

Among 7 men starting weight loss treatment there was 1 (14.3%) subject with a high level of body size dissatisfaction, 1 (14.3%) moderate and 5 (71.4%) low; and among not seeking treatment for obesity 2 (3.6%), 15 (27.3%) and 38 (69.1%), respectively (Fig. 3). There was a significant difference in the distribution of body size dissatisfaction in women (χ^2^ = 7.0; *p* < 0.05), but not in men (χ^2^ = 1.9; *p* = 0.47).

**Fig. 4:**
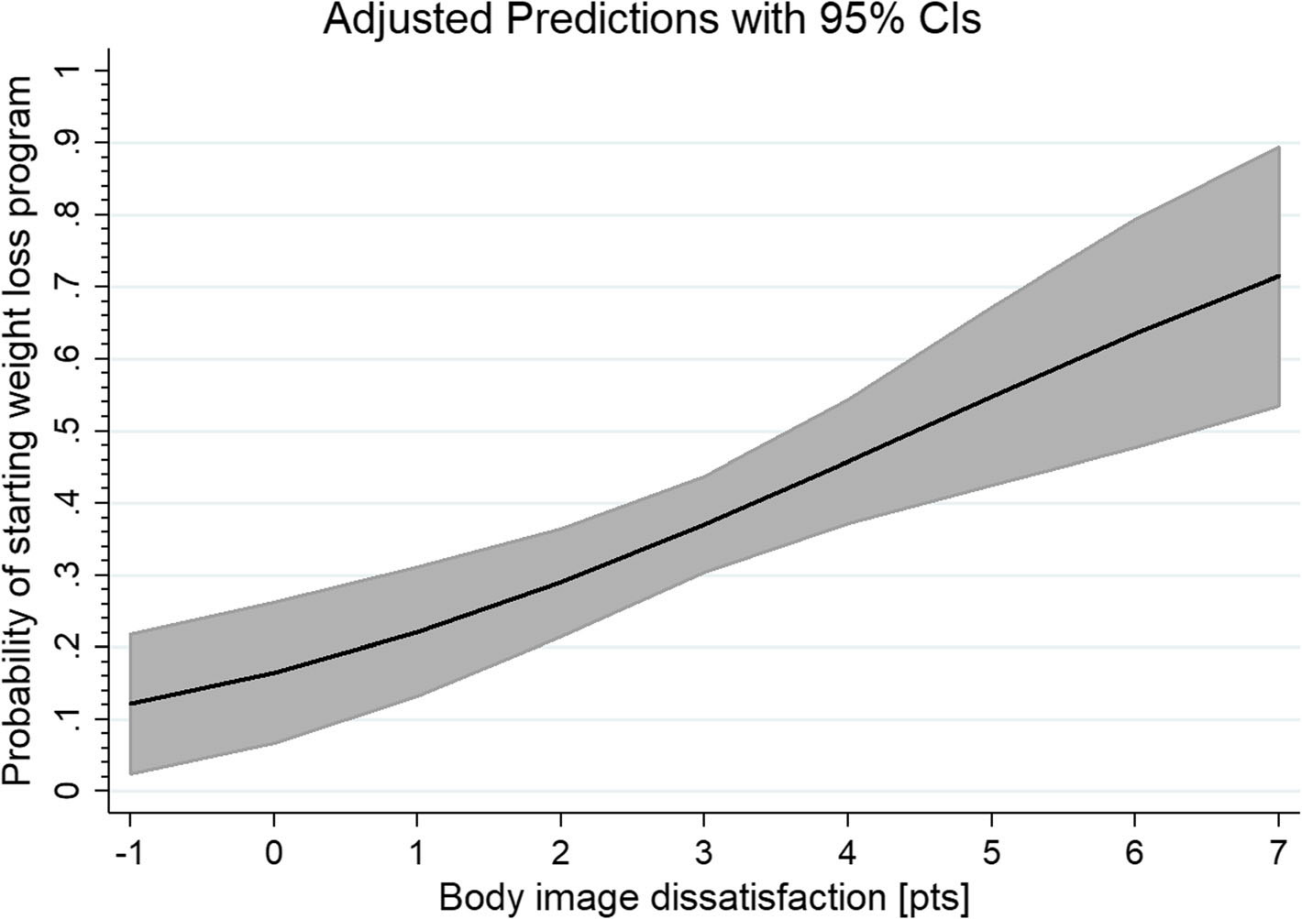
Probability of starting weight loss program according to body image dissatisfaction in analyzed group of subjects. Grey area depicts 95% confidence interval

A multiple stepwise backward logistic regression analysis, adjusted to gender, revealed that only body image dissatisfaction was significantly associated with uptake of the treatment (OR = 1.44; 95% CI: 1.19–1.73; *p* < 0.001) - Fig. 4. The goodness-of-fit was established (*p* = 0.65).

### Body size dissatisfaction and the adherence to the obesity treatment program and its effectiveness

Among the treatment group, 54% of patients completed the program (adherence rate defined as the number of completed patient’ check-up / the number of total scheduled check-up > 80%). The level of body size dissatisfaction did not influence the adherence to the group obesity treatment program and its effectiveness measured by obtained weight loss.

## Discussion

Our study has shown that patients with obesity starting obesity treatment more often have a high level of body size dissatisfaction than volunteers with obesity not seeking treatment for obesity. This confirms the initial hypothesis of psychological heterogeneity of subjects living with obesity by Fitzgibbon [8] and is in line with previous findings showing a higher frequency of depression symptoms in people with obesity starting obesity treatment than in volunteers with obesity not seeking treatment for obesity [9]. It should be noted that adjustment for age and BMI values did not influence obtained results. This is opposed to studies showing that age and BMI are important factors that modified body image [10–17, 22]. One possible reason for this may be related to the tools used for evaluation. Also Bianciardi et al. [23], in line with our results, did not find a correlation between body image dissatisfaction and BMI in patients with extreme obesity. Other, previous studies which reported relations between BMI and body image dissatisfaction were mostly based on observation of body image aspects before and after weight reduction [22] or the comparison of patients with obesity with normal-weight individuals [24]. The lack of the impact of age on the observed association may be the result of relative age-homogeneity of the study group, as the presented study was based on middle-aged respondents. Besides, some studies suggest that age does not affect body image disturbances and that body image remains stable throughout the life span, especially in women [13]. Younger people seem to experience greater “social pressure” [12]. Although older people, especially women, still pay attention to their appearance [31], even though they may not feel the same social pressure as younger people to be slim and beautiful [32].

In particular, the observed difference in the distribution of body size dissatisfaction was present among women. It should be indicated that the number of men in the study subgroups was small, which is in general a limitation of studies on obesity [33]. As already mentioned, it seems that women are much more likely to be dissatisfied with body image, and this dissatisfaction is additionally more intense than in men. It may be caused by changes in the secretion of hormones in women during puberty, phases of the menstrual cycle, pregnancy and menopause [13]. Moreover, mass media create a more demanding external image concerning women and associate their appearance with specific character traits such as ambition and discipline [34, 35]. In turn, men, seem to pay less attention to their body image, although this image seems to have gained importance in recent years [36]. Furthermore, men seem to be less involved in the image of their own body as a whole, but they are particularly focused on the muscles, with which they are most often dissatisfied [37]. The above observations on the less common occurrence of body size dissatisfaction among men combined with the presented results may partially explain the common difficulties in recruiting men to various studies on obesity.

Our results suggest that in some subjects’ body size dissatisfaction, among other factors, may affect the decision to start obesity treatment. This rather positive role stays in contrast to studies showing that body image dissatisfaction has a negative impact on healthy behaviour. It was observed that lower body image satisfaction is related to lower physical activity both in women and men. Furthermore, it was shown that women starting physical training for body image reasons are less physically active than those doing this for pleasure or good feeling [38]. However, the data on the role of body image disturbances on this behaviour was mainly based on normal-weight respondents. In our study, high body image dissatisfaction promoted the decision to start obesity treatment, which suggests its rather positive impact. It seems that subjects with obesity starting the obesity treatment programs should be assessed on body image satisfaction. In the case of positive finding professional body image interventions seems to be inevitable. The eventual parallel introduction of them to obesity treatment programs needs further studies. Of note, it seems reasonable to deepen the comprehension of body image dissatisfaction, particularly in women and improve the therapeutic strategies and ameliorate treatments results [39].

Obesity still goes largely untreated. Public health campaigns are needed to increase awareness of the psychosocial impairment associated with the excessive body weight. Particularly, it can be very important for clinicians to approach patients with obesity, neither seeking obesity treatment nor perceiving their obesity per se as a health problem. Special efforts to encourage them to start obesity treatment would be of benefit. Further, personal goals setting, such as body image improvement, may help strengthen the doctor-patient relationship and corroborate therapeutic strategies [40–42]. On the other hand, presented results can also imply that clinicians should screen female patients with obesity seeking treatment for obesity for body image disturbances. In case of a positive result, refer them to psychological intervention.

The main limitations of the presented study are the small number of men in the study group, especially in the group starting obesity treatment and assessment only attitudinal aspects of body image. Although various techniques have been developed to assess body image disturbance, mostly in patients with eating disorders [24, 43–45], Stunkard’s Figure Rating Scale is one of the oldest ones, very simple in construction. Perhaps, it is not as reliable as a clinical interview or some other questionnaires, but quick and easy to use. Due to the use of the silhouettes, it does not need adaptation to the native language of studied subjects and can be also used in patients with low literacy. It seems that it can be very easily administered in clinical practice worldwide. Moreover, the recruitment strategy for the study appears to have a limitation. Firstly, it cannot be ruled out that patients starting obesity treatment, despite applying to the program voluntarily, were not persuaded to do so by, for example, their partners or other family members. Secondly, creating a control group of unmotivated to act, generally seems to be a difficult task. Studies on the motivation to lose weight alone, as well as in obesity treatment programs in both adolescents [46] and adults [47] are largely devoid of control groups. In this sense, the proposed recruitment strategy in the presented study on motivation appears to be innovative, however, it requires further improvements. Another limitation is the lack of data about self-weighing (never, every day, week, month). There is evidence to suggest self-weighing may have a negative effect on mood and body image after a short period of self-weighing [48]. What is more, following a full intervention, a positive correlation between self-weighing and body-related attitudes was observed among patients with obesity [49]. However, the relationship between self-weighing and body weight, to the best of our knowledge, is still an area for future investigation. Only one factor potentially influencing the decision to start treatment was taken into account in our study. There is a great need to create a more detailed profile of people with BMI ≥ 30 kg/m^2^, who are not prone to seek obesity treatment. As noted earlier, numerous internal and external factors seem to affect this decision. Therefore a more multidimensional approach is needed.

## Conclusion

Patients with obesity, especially women, seeking obesity treatment more often represent a high level of body size dissatisfaction than volunteers with obesity not seeking treatment for obesity. This implies the need for public health campaigns to address negative attitudes or misconceptions about obesity and its treatment. Placing more attention and emphasis on body size dissatisfaction in more vulnerable women with obesity may help to define personal motivations and goals, strengthen the doctor-patient relationship and better adapt therapeutic strategies.

## Data Availability

The datasets used and /or analyzed during the current study are available from the corresponding author on reasonable request.

## Abbreviations

BMI: Body Mass Index
FRS: Figure Rating Scale
WHO: World Health Organization
